# Integration of plasma and electrocatalysis to synthesize cyclohexanone oxime under ambient conditions using air as a nitrogen source†

**DOI:** 10.1039/d3sc02871b

**Published:** 2023-10-30

**Authors:** Shunhan Jia, Xingxing Tan, Limin Wu, Xiaodong Ma, Libing Zhang, Jiaqi Feng, Liang Xu, Xinning Song, Qinggong Zhu, Xinchen Kang, Xiaofu Sun, Buxing Han

**Affiliations:** a Beijing National Laboratory for Molecular Sciences, CAS Laboratory of Colloid and Interface and Thermodynamics, CAS Research/Education Center for Excellence in Molecular Sciences, Center for Carbon Neutral Chemistry, Institute of Chemistry, Chinese Academy of Sciences Beijing 100190 China sunxiaofu@iccas.ac.cn hanbx@iccas.ac.cn; b School of Chemical Sciences, University of Chinese Academy of Sciences Beijing 100049 China; c State Key Laboratory of Organic-Inorganic Composites, College of Chemical Engineering, Beijing University of Chemical Technology Beijing 100029 China; d Shanghai Key Laboratory of Green Chemistry and Chemical Processes, School of Chemistry and Molecular Engineering, East China Normal University Shanghai 200062 China

## Abstract

Direct fixation of N_2_ to N-containing value-added chemicals is a promising pathway for sustainable chemical manufacturing. There is extensive demand for cyclohexanone oxime because it is the essential feedstock of Nylon 6. Currently, cyclohexanone oxime is synthesized under harsh conditions that consume a considerable amount of energy. Herein, we report a novel approach to synthesize cyclohexanone oxime by *in situ* NO_3_^−^ generation from air under ambient conditions. This process was carried out through an integrated strategy including plasma-assisted air-to-NO_*x*_ and co-electrolysis of NO_*x*_ and cyclohexanone. A high rate of cyclohexanone oxime formation at 20.1 mg h^−1^ cm^−2^ and a corresponding faradaic efficiency (FE) of 51.4% was achieved over a Cu/TiO_2_ catalyst, and the selectivity of cyclohexanone oxime was >99.9% on the basis of cyclohexanone. The C–N bond formation mechanism was examined by *in situ* experiments and theoretical calculations, which showed that cyclohexanone oxime forms through the reaction between an NH_2_OH intermediate and cyclohexanone.

## Abstract 2

We propose a sustainable strategy to efficiently synthesize cyclohexanone oxime using air as the N source. The process is carried out through an integrated strategy including plasma-assisted air-to-NO_*x*_ and co-electrolysis of NO_*x*_ and cyclohexanone.

## Graphical abstract

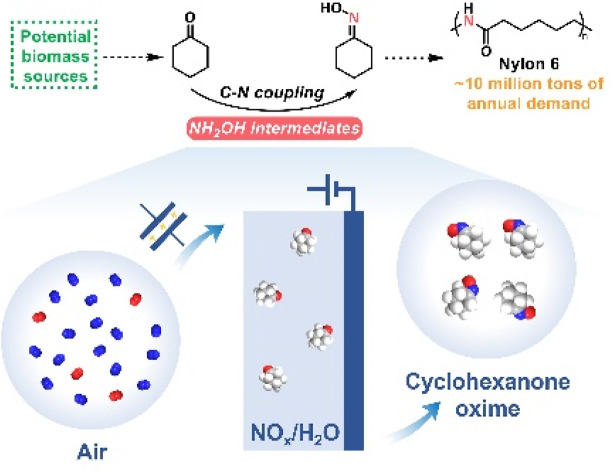

## Introduction

Dinitrogen (N_2_) constitutes approximately 80% of the air, and is an inexhaustible nitrogen source that can be used to produce N-containing chemicals with a wide range of applications.^1,2^ However, because of the ultra-inert N≡N triple bonds, the kinetics are sluggish and the thermodynamics are limited for the conversion of N_2_.^3–5^ Over the past few decades, researchers were mainly focused on the fixation of N_2_ to NH_3_ as a feedstock that would be further processed into various organonitrogen chemicals through reductive amination, oxidative cyanation, and ammoxidation.^6–8^ Industrial NH_3_ production through the traditional Haber–Bosch process consumes large amounts of energy and emits high levels of CO_2_, and therefore cannot meet the demands for carbon neutrality or sustainability goals.^9–11^ As an alternative route, the direct utilization of N_2_ to construct C–N bonds under mild conditions is a promising strategy for the production of value-added chemicals and the decoupling of chemical manufacturing from fossil fuel energy, but it is a challenge.^12–14^

There is great interest in the use of the electrochemical N_2_ reduction reaction (NRR) to produce NH_3_ because of the utilization of renewable electricity and protons directly from water.^15–17^ The electrochemical construction of the C–N bond *via* the coupling of CO_2_ and N_2_ has also been considered to produce some organonitrogen chemicals, such as urea.^18,19^ However, the reported reactions are very limited, and they are hindered by low reactivity and selectivity due to low N_2_ solubility, difficult N_2_ activation, and undesired hydrogen evolution reaction (HER).

Based on the occurrence of lightning in nature, air plasma oxidation offers an effective method for N_2_ activation. Air can be converted into reactive NO_*x*_ or NO_*x*_^−^, which can be used as a N source in electrolysis.^20,21^ The C–N coupling would be achieved by the *in situ* generation of nucleophilic N-containing intermediates followed by their coupling with carbon substrates or intermediates as the electrophile.^22,23^

**Scheme 1:**
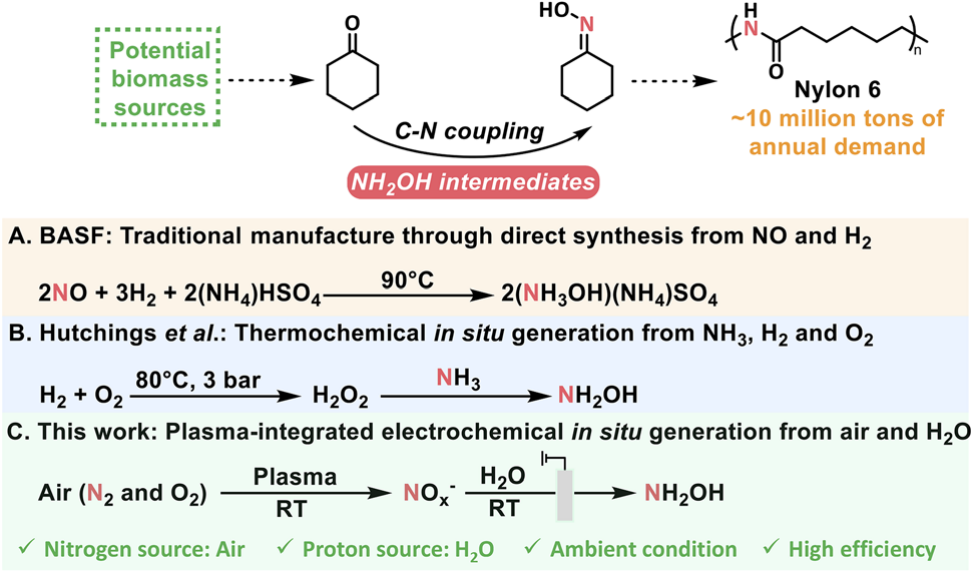
Schematic diagram of different production routes for cyclohexanone oxime.

Cyclohexanone oxime is the essential feedstock of Nylon 6, with worldwide annual demand of approximately 10 million tons and an approximate global market size of USD 25 billion, and is currently produced through the coupling between cyclohexanone and NH_2_OH intermediates from unsustainable (NH_3_OH)(NH_4_)SO_4_ (Scheme 1).^24–26^ Therefore, optimization of N sources for NH_2_OH intermediates is urgently needed for cyclohexanone oxime production. Researchers have recently explored the synthesis of cyclohexanone oxime using *in situ*-generated H_2_O_2_ to produce NH_2_OH intermediates from NH_3_.^8^ Furthermore, the direct use of N_2_*via* air plasma oxidation integrated with electroreduction may result in the formation of cyclohexanone oxime under ambient conditions.

Herein, we report the first work to efficiently synthesize cyclohexanone oxime using air as the nitrogen source by combining plasma-assisted air-to-NO_*x*_ and co-electrolysis of NO_*x*_ and cyclohexanone. A faradaic efficiency (FE) of 51.4% was achieved for cyclohexanone oxime with a corresponding formation rate of 20.1 mg h^−1^ cm^−2^ and remarkable catalyst (Cu/TiO_2_) recyclability. The superior performance can be attributed to Cu sites supported on TiO_2_ with a stable oxidation state. In addition, the reaction pathway was studied based on *in situ* experiments and theoretical calculations.

## Results and discussion

**Fig. 1:**
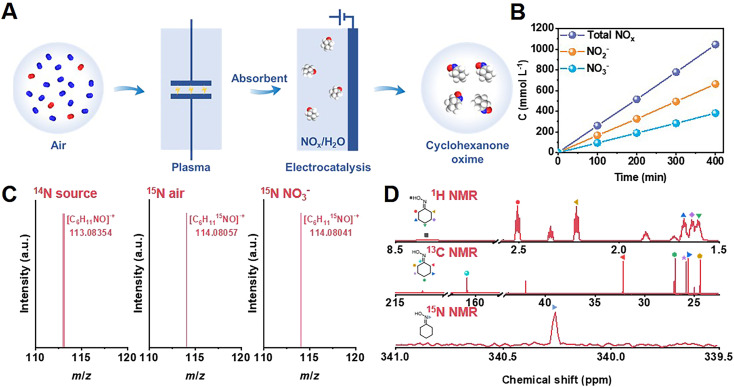
The integration system for cyclohexanone oxime synthesis. (A) Schematic illustration of the synthesis of cyclohexanone oxime with air as nitrogen source. (B) The concentration of NO_*x*_^−^ species under air activation. (C) GC-MS and (D) ^1^H NMR, ^13^C NMR, and ^15^N NMR detection of cyclohexanone oxime.

As shown in Fig. 1A, as a proof-of-concept, we synthesized cyclohexanone oxime through the coupling of plasma air activation and NO_*x*_ electroreduction with cyclohexanone (Fig. S1†). A model air mixture of N_2_/O_2_ (v/v = 4/1) was used as the nitrogen source. The reaction between N_2_ and O_2_ activated by plasma produced NO_*x*_, which was absorbed by 1 mol L^−1^ NaOH aqueous solution, and the as-obtained solution was directly used as an electrolyte for further electrosynthesis (Fig. S2 and S3†). This process holds the potential to block the interference of trace H_2_O and CO_2_ from real air. The concentration of NO_3_^−^ and NO_2_^−^ was quantified by UV-Vis spectroscopy measurements (Fig. S4†). Fig. 1B shows that the yield of NO_*x*_ linearly increased over the plasma activation time, in which the concentration of NO_2_^−^ was approximately 2 times that of NO_3_^−^. A high total NO_*x*_ concentration of approximately 1 mol L^−1^ was achieved after 400 min of plasma activation.

In the following electrosynthesis process, the electroreduction of NO_3_^−^ with cyclohexanone was initially verified as a model reaction in a flow cell with 1 mol L^−1^ NaNO_3_ and 50 mmol L^−1^ cyclohexanone aqueous solution as the catholyte, and 1 mol L^−1^ NaOH as the anolyte. Copper-based catalysts were selected due to their exceptional performance in nitrate reduction, as described in recent reports.^27–31^ Cu/TiO_2_ catalysts loaded onto porous carbon paper served as the cathode, and Ni foam was used as the anode.

The obtained catalysts contained 0.3, 0.6, and 0.9 wt% Cu, as determined by inductively coupled plasma optical emission spectroscopy (ICP-OES). We denoted these catalysts as 0.3% Cu/TiO_2_, 0.6% Cu/TiO_2_, and 0.9% Cu/TiO_2_ for clarity. H_2_ was the only gas product, and cyclohexanone oxime was the only organic product during electrolysis quantified by gas chromatography (GC). Colorimetric methods were used to study the aqueous byproducts, including NO_2_^−^ and NH_3_ (Fig. S4 and S5†). We also identified cyclohexanone oxime *via* GC-mass spectrometry (GC-MS) and nuclear magnetic resonance (NMR) (Fig. S6–S10†). As shown in Fig. 1C, the molecular weight of 113.08354 provided by high-resolution GC-MS indicated that the molecular formula of the product is C_6_H_11_NO.

Using model air with ^15^N_2_ gas and Na^15^NO_3_ as the reactant, the molecular ion peak of the product at 114.08057 and 114.08041 matched the calculated weight of 114.08055 for C_6_H_11_^15^NO, which verified that the origin of nitrogen in cyclohexanone oxime was NO_3_^−^*in situ* generated from air. Moreover, all the peaks in the ^1^H NMR, ^13^C NMR, and ^15^N NMR (conducted with the ^15^N-labeled product) spectra were well matched with the standard samples (Fig. 1D and S9†). These results confirmed the formation of cyclohexanone oxime, with NO_3_^−^ as the nitrogen source.

**Fig. 2:**
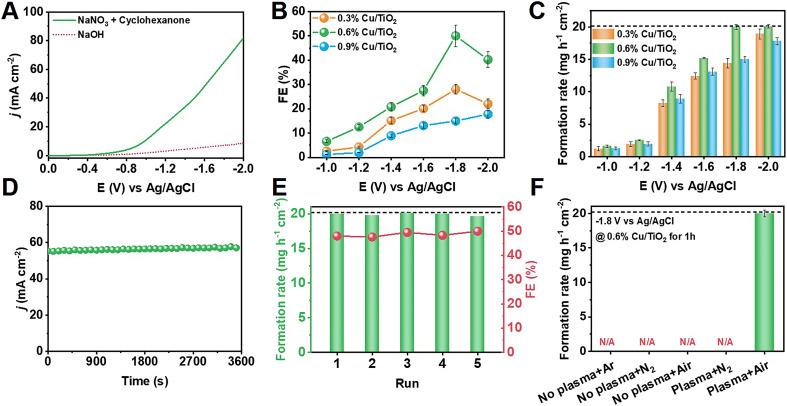
Electrochemistry study of cyclohexanone synthesis. (A) LSV curves of 0.6% Cu/TiO_2_ catalysts in different electrolyte. Potential-dependent (B) FE and (C) formation rate of cyclohexanone oxime production on different Cu-loaded TiO_2_ catalysts. (D) Chronoamperometry curves of 0.6% Cu/TiO_2_ under optimal conditions for cyclohexanone oxime synthesis. (E) Recyclability of 0.6% Cu/TiO_2_ electrocatalysts. (The dotted line shows the formation rate of cyclohexanone at 100% conversion.) (F) The formation rate of cyclohexanone oxime with different gas feed and plasma conditions.

Fig. 2A displays the linear sweep voltammetry (LSV) results for 0.6% Cu/TiO_2_ in electrolyte with and without NaNO_3_ plus cyclohexanone substrates. After the addition of the substrates, the sharpness of the increase in the current density (*j*) was greater than that of the initial NaOH electrolyte, which demonstrated that the electrosynthesis of cyclohexanone was more kinetically favorable than the HER. Electrochemical performance tests were carried out in the range of −1.0 V to −2.0 V *versus* Ag/AgCl, and the electrochemical performance of cyclohexanone oxime formation on Cu/TiO_2_ is shown in Fig. 2B and C. The reactions under all the applied potentials presented a combined FE of approximately 100% for all products. A maximum cyclohexanone oxime FE of 50.0% and a corresponding formation rate of 20.1 mg h^−1^ cm^−2^ was achieved at −1.8 V over 0.6% Cu/TiO_2_.

At low potentials, NO_2_^−^ was the main byproduct, which could be attributed to the hindered reductive hydrogenation of NO_3_^−^. Once the potential increased so that it was more negative than −1.8 V, the enhancement of the HER and the further electroreduction of NH_2_OH intermediates led to a decrease in the selectivity for cyclohexanone oxime (Fig. S11†). Intriguingly, the byproducts generated during the electrolysis process, specifically NH_3_ and H_2_, hold significant value as chemicals with substantial potential for further utilization. Under optimal conditions, the FE of NH_3_ and H_2_ were 21.6% and 16.4%, respectively. Similar trends were found for the Cu/TiO_2_ catalysts with various Cu loadings. In addition, as shown in Fig. 2D, the corresponding chronoamperometry curve of 0.6% Cu/TiO_2_ catalysts at −0.8 V remained stable during electrosynthesis.

We further studied the influence of substrate concentrations on the electrochemical performance by varying the molar concentrations of cyclohexanone and NaNO_3_ in the electrolytes at the applied potential of −1.8 V *vs.* Ag/AgCl (Fig. S12†). This indicated that a higher cyclohexanone and NO_3_^−^ concentration promoted the formation of cyclohexanone oxime. However, when the concentration of cyclohexanone was greater than 50 mmol L^−1^, there was a significant decrease in the cyclohexanone oxime FE and formation rate due to the phase separation in the catholyte. The increase in the NO_3_^−^ concentration up to 2 mol L^−1^ strengthened the side reaction of NH_3_ formation, which was unsuitable for the formation of NH_2_OH intermediates and cyclohexanone oxime. Consequently, the optimized substrate concentration was found to be 50 mmol L^−1^ cyclohexanone and 1 mol L^−1^ NaNO_3_ for cyclohexanone oxime production.

The TiO_2_ support played a crucial role in the improvement of cyclohexanone oxime formation. Fig. S13† shows that the catalytic performance of the bulk TiO_2_ was very poor. For comparison, carbon black was also used as a support for Cu loading, but the as-prepared Cu/carbon black catalyst exhibited a maximum cyclohexanone oxime FE of only 8.4% with a formation rate of 2.5 mg h^−1^ cm^−2^. Therefore, the optimized catalytic activity could be ascribed to the cooperation between Cu and the TiO_2_ support.

The stability of the 0.6% Cu/TiO_2_ catalyst was also studied at −1.8 V. Fig. 2E shows that there was no notable change in the cyclohexanone oxime FE or formation rate during 5 successive cycles, which was indicative of prominent recyclability and is important for practical applications.

After establishing the baseline of NO_3_^−^ electrolysis activity, NO_*x*_ obtained from plasma air activation was fed together with cyclohexanone in the electrosynthesis process. Fig. 2F shows that the integrated system provided cyclohexanone oxime at a formation rate of 20.1 mg h^−1^ cm^−2^ using air as the nitrogen source. No cyclohexanone oxime was detected in the absence of either plasma, N_2_, or O_2_, indicating the necessity of air as the nitrogen source under plasma oxidation for cyclohexanone oxime production. It was therefore concluded that the two-step integration of plasma air activation with electrocatalytic reduction is practically feasible for highly efficient and selective nitrogen synthesis of cyclohexanone oxime under ambient conditions.

**Fig. 3:**
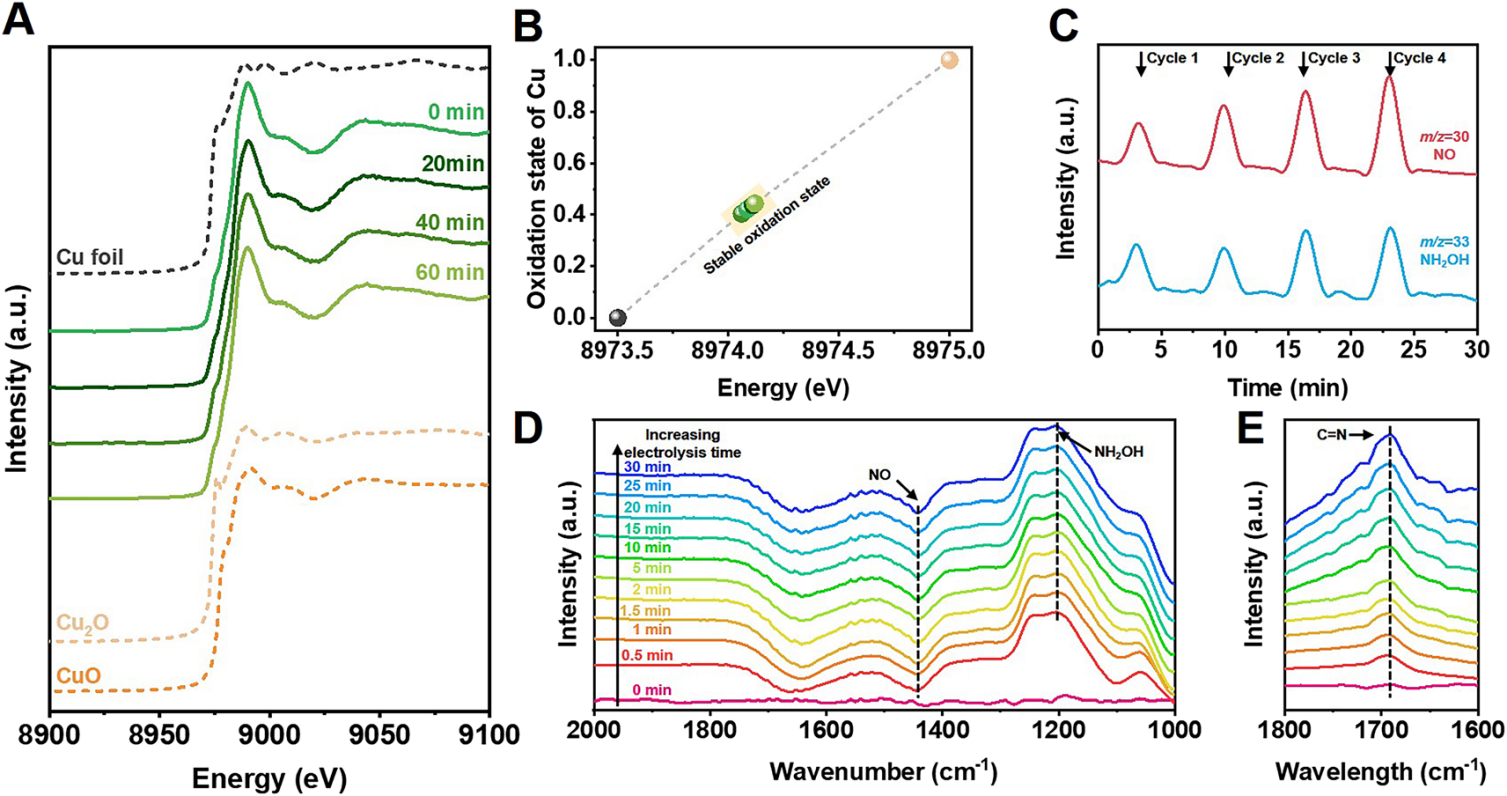
*In situ* study. (A) XANES spectra at Cu K-edge and (B) Cu oxidation state of 0.6% Cu/TiO_2_ measured at different electrolysis times during cyclohexanone oxime synthesis. (C) Online DEMS graph of NO and NH_2_OH intermediates. *In situ* FTIR spectra of electrochemical cyclohexanone synthesis conducted at prolonged electrolysis time on 0.6% Cu/TiO_2_ in (D) H_2_O and (E) D_2_O solvent.

Structural and morphological measurements clarified that Cu nanoparticles were uniformly loaded on TiO_2_ in the Cu/TiO_2_ catalysts (Fig. S14–S22†). *In situ* X-ray absorption near-edge spectroscopy (XANES) was conducted to further investigate the electronic structure of the catalysts during cyclohexanone oxime synthesis. Fig. 3A shows that an initial 0.6% Cu/TiO_2_ catalyst exhibited an absorption edge between that of Cu foil and Cu_2_O samples, indicating that the oxidation state of Cu was between 0 and +1. We further acquired the Cu average oxidation state as a function of the Cu K-edge energy shift, and the value was 0.42 during the *in situ* measurements (Fig. 3B). The value did not change over electrolysis time, indicating the excellent stability of the Cu active sites during electrolysis. The results indicated that a stable slightly positive oxidation state for Cu was beneficial for the reaction. In Fig. S23 and S24,† no obvious changes were observed in the structure or morphology of the catalyst after electrolysis, further indicating the stability of the catalyst.

Further study was conducted to reveal the possible reaction pathway. Online differential electrochemical mass spectrometry (DEMS, Fig. S25†) was used to detect the *m/z* signals of 30 and 33, as shown in Fig. 3C, which corresponded to the NO and NH_2_OH intermediates, respectively, during NO_3_^−^ reduction over 0.6% Cu/TiO_2_ catalyst. The weak signal of NH_2_OH indicated that the generated NH_2_OH spontaneously coupled with cyclohexanone to produce oxime products and could not remain in the bulk electrolyte. Signals of other byproducts were also detected, as shown in Fig. S26.†*In situ* Fourier transform infrared (FTIR) spectroscopy (Fig. S27†) also confirmed the possible involvement of NO and NH_2_OH signals at 1442 and 1204 cm^−1^ in Fig. 3D on the surface of the 0.6% Cu/TiO_2_ catalyst during electrosynthesis.^32–34^ Additionally, when using D_2_O as the electrolyte solvent, an obvious peak at 1689 cm^−1^ was observed, as shown in Fig. 3E, indicating the formation of C=N bonds in cyclohexanone oxime molecules that was not observed in H_2_O solvent because of the interference of O–H bending (1645 cm^−1^).

Systematic control experiments were performed to study the reaction pathways of the electrosynthesis of cyclohexanone oxime (Table S1†). No cyclohexanone oxime was detected when there was no cyclohexanone or NO_3_^−^ in the electrolyte (entries 1–3). We also used NO_2_^−^, NO, and NH_2_OH as the feedstock, which were formerly identified as the intermediates (entries 4–6). All three entries produced cyclohexanone oxime products. In addition, the C–N coupling between cyclohexanone and NH_2_OH was proved to be spontaneous, and occurred without electrolysis (entries 6–7).

The discernible fluctuations in the FE, notably when feeding with NO, were ascribable to the limited solubility of NO gas in aqueous solutions, leading to heightened hydrogen evolution side reactions. Notwithstanding these disparities in the FE, the rate of cyclohexanone oxime formation continued to exhibit a noteworthy level of consistency across all nitrogenous substrates. Therefore, the above results present the possible reaction pathways of NO_3_^−^ to NH_2_OH intermediates through NO_3_^−^ → NO_2_^−^ → NO → NH_2_OH progression, followed by the nonelectrochemical spontaneous condensation of NH_2_OH and cyclohexanone to produce cyclohexanone oxime.

**Fig. 4:**
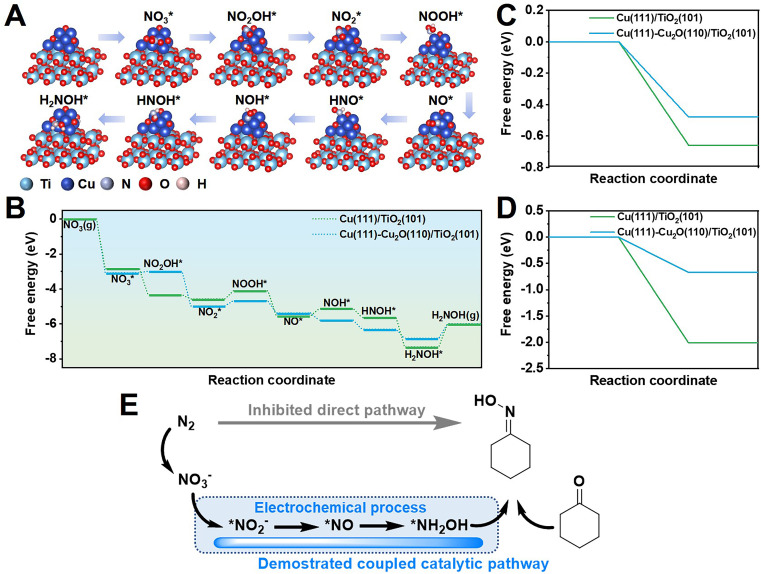
Theoretical calculations. (A) Catalytic pathway from NO_3_^−^ to NH_2_OH on Cu/TiO_2_ catalyst according to the optimized configurations with adsorbed intermediates. Cyan, blue, purple, red, and pink balls denote Ti, Cu, N, O, and H, respectively. (B) Gibbs free energy diagrams of NH_2_OH generation from NO_3_^−^ on different models. (C) Calculated free energy diagram for (C) cyclohexanone* and (D) cyclohexanone oxime* adsorption on different TiO_2_-loaded Cu catalytic sites. (E) The proposed pathway of NH_2_OH generation and cyclohexanone oxime production.

Furthermore, density functional theory (DFT) calculations were conducted to study the reaction pathway (Fig. 4A and S28†). According to high-resolution transmission electron microscopy (HRTEM) images and X-ray diffraction (XRD) patterns, Cu(111) and Cu(111)-Cu_2_O(110) heterostructures loaded on anatase phase TiO_2_(101) were used as the catalyst models. A free energy diagram of NO_3_^−^-to-NH_2_OH is presented in Fig. 4B. NO_3_^−^ was chemically absorbed on the catalyst to initially form 
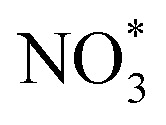
 with a decrease in free energy. Then, the deoxygenation and hydrogenation steps were continuously proceeded by H^+^/e^−^ transfer to form 
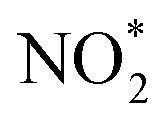
 and NO*. The conversion of 
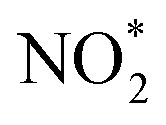
 to NOOH* was endergonic and acted as the rate-determining step (RDS), which was consistent with the detection of NO_2_^−^ byproducts during electrolysis. Subsequently, the NO* intermediate was gradually converted to NOH*, NHOH*, and finally to NH_2_OH* under hydrogenation.

The high selectivity of >99.9% is an obvious advantage of cyclohexanone oxime production through an electrocatalysis strategy.^8,35,36^ Interestingly, the reduction of cyclohexanone and cyclohexanone oxime were not detected as side reactions because no GC-MS peaks of cyclohexanol or cyclohexylamine appeared in Fig. S6A.† The reduction of excess NO_*x*_ substrates as well as a coherent base environment (pH 13.4) of the electrolyte hindered the reduction of organic molecules.^12^ Additionally, DFT study revealed the limitation of reduction of cyclohexanone (Fig. 4C) and cyclohexanone oxime (Fig. 4D). The calculated free energy of the absorption of cyclohexanone and cyclohexanone oxime on Cu(111)-Cu_2_O(110) models was found to be lower than that on the Cu(111) structure, which implies that the side reactions were hindered during efficient production of cyclohexanone oxime on Cu/TiO_2_ catalysts. As discussed above, the pathway of cyclohexanone is presented in Fig. 4E. NO_*x*_ substrates from air oxidation produced NH_2_OH through electrocatalysis, followed by its non-electrochemical coupling with cyclohexanone to produce oxime.

The universality of our method was further confirmed by successfully converting a range of aldehydes and ketones in addition to cyclohexanone (*e.g.*, benzaldehyde, acetone, cyclopentanone, and diethyl ketone) into oximes with high FE and selectivity under optimal conditions (Table S2†). This highlights the broad applicability of the electrocatalytic strategy to synthesize oxime, and affirms our proposed reaction pathway.

## Conclusions

We propose a sustainable strategy to efficiently synthesize value-added cyclohexanone oxime using air as a nitrogen source, which includes plasma-assisted air-to-NO_*x*_ and co-electrolysis of NO_*x*_ and cyclohexanone. The highest performance was provided by 0.6% Cu/TiO_2_, which produced cyclohexanone at a yield rate of 20.1 mg h^−1^ cm^−2^ and an FE of 51.4%. The selectivity of cyclohexanone oxime was >99.9% on the basis of cyclohexanone. Detailed study indicated that the optimized catalytic activity was ascribed to Cu sites supported on TiO_2_ with a stable oxidation state.

Further *in situ* characterizations combined with DFT calculations revealed the possible pathway of NO_3_^−^ → NO_2_^−^ → NO → NH_2_OH → cyclohexanone oxime. Together with transformation routes reported by our group, including the production of phenol using lignin and selective phenol-to-cyclohexanone conversion, this work provides a rational pathway to the synthesis of cyclohexanone oxime with biomass, an air nitrogen source, and green solvent.^37–39^ It will also inspire innovative routes to the carbon-natural chemical manufacturing of industrially important fine C–N chemicals from renewable carbon sources and nitrogen using clean energy.

## Data availability

All experimental data is available in the ESI.†

## Author contributions

S. H. J., X. F. S., and B. X. H. proposed the project, designed the experiments, and wrote the manuscript. S. H. J. performed all the experiments. X. X. T., L. M. W., and X. D. M. performed the analysis of the experimental data. L. B. Z., J. Q. F., L. X., and X. N. S. conducted portions of the characterization study. Q. G. Z. and X. C. K. participated in discussions. X. F. S. and B. X. H. supervised the entire project.

## Conflicts of interest

There are no conflicts to declare.

## Supplementary Material

SC-014-D3SC02871B-s001
