# Supplementary material for: Integration of plasma and electrocatalysis to synthesize cyclohexanone oxime under ambient conditions using air as a nitrogen source
Source: Chem Sci. 2023 Oct 30;14(45):13198–204. doi: 10.1039/d3sc02871b (PMC10664508; doi:10.1039/d3sc02871b)
Supplement: SC-014-D3SC02871B-s001 [file SC-014-D3SC02871B-s001.pdf]

Electronic Supplementary Material (ESI) for Chemical Science.
This journal is © The Royal Society of Chemistry 2023

Electronic Supplementary Information

Synthesis of Cyclohexanone Oxime using Air as Nitrogen Source under Ambient Conditions by Integration of Plasma and Electrocatalysis

Shunhan Jia,[a,b] Xingxing Tan,[a], Limin Wu,[a,b] Xiaodong Ma,[a] Libing Zhang,[a,b] Jiaqi Feng,[a] Liang Xu,[a,c] Xinning Song,[a,b] Qinggong Zhu,[a,b] Xinchen Kang,[a,b] Xiaofu Sun,*[a,b] and Buxing Han*[a,b,d]

[a.] *Beijing National Laboratory for Molecular Sciences, CAS Laboratory of Colloid and Interface and Thermodynamics, CAS Research/Education Center for Excellence in Molecular Sciences, Center for Carbon Neutral Chemistry, Institute of Chemistry, Chinese Academy of Sciences, Beijing 100190, China. E-mail: sunxiaofu@iccas.ac.cn (X. Sun), hanbx@iccas.ac.cn (B. Han)*

[b.] *School of Chemical Sciences, University of Chinese Academy of Sciences, Beijing 100049, China*

[c.] *State Key Laboratory of Organic-Inorganic Composites, College of Chemical Engineering, Beijing University of Chemical Technology, Beijing 100029, China*

[d.] *Shanghai Key Laboratory of Green Chemistry and Chemical Processes, School of Chemistry and Molecular Engineering, East China Normal University, Shanghai 200062, China*

Experimental Procedures

Materials

Cyclohexanone (99%), cyclohexanone oxime (99%), n-dodecane (99%), sodium nitrate ($NaNO_3$, 99%), sodium nitrite ($NaNO_2$, 97%), hydroxylamine sulfate, [$(NH_2OH)_2 \cdot H_2SO_4$, 99%], deuterium oxide (D_2O, 99.8%), Chloroform-d ($CdCl_3$, 100% isotopic, contains 0.03% v/v TMS) and sodium borohydride ($NaBH_4$, 98%) were purchased from Alfa Aesar Co., Ltd. ^{15}N-Hydroxylamine sulfate [$(NH_2OH)_2 \cdot H_2SO_4$, 95%] was purchased from Sigma-Aldrich LLC. Copper(II) nitrate trihydrate, [$Cu(NO_3)_2 \cdot 3H_2O$, 99%], and titanium(IV) oxide (TiO_2, anatase powder, 98%) were obtained from Acros Organics, Thermo Fisher Scientific Inc. Nickel foam (NF, 99.99%), 4-aminobenzenesulfonamide (99%), N-(1-naphthyl) ethylenediamine dihydrochloride (98%), sodium salicylate (99.5%), sodium hypochlorite (active chlorine>10%), sodium nitroferricyanide dihydrate (99%) were provided by Innochem (Beijing) Technology Co., Ltd. Hydrochloric acid (HCl, 36.0~38.0%) was obtained from Sinopharm Chemical Reagent Co., Ltd. Ethanol (99.8%), ethyl acetate (EA, 99.8%), acetone (99.8%), and isopropanol (IPA, 99.8%) was purchased from Concord Technology (Tianjin) Co., Ltd. Deionized (DI) water was used in the experiments. All the chemicals were used as received without further purification.

Preparation of TiO_2 electrocatalysts

100 mg of commercial TiO_2 nanoparticles were heated at 500°C for 2 h (heating rate: 5 °C min^{-1}) under the 10% H_2/90% Ar atmosphere after purging for 20 min to remove the air. After cooling down to room temperature, TiO_2 was collected for further use.

Preparation of $Cu_{0.6\%}/TiO_2$ electrocatalysts

30 mg of TiO_2 sample was added to 20 mL of DI water followed by ultrasonic treatment for 20 min. After adding 150 µL of $Cu(NO_3)_2 \cdot 3H_2O$ (5 mg mL^{-1}) aqueous solution followed by stirring at 1000 rpm for 30 min, 5 ml $NaBH_4$ (10 mg ml^{-1}) aqueous solution was added slowly to the solution at the rate of ca. 200 µL min^{-1} with a syringe infusion pump. After another 20 min string, the precipitate separated by centrifuge (8000rpm, 3 min) was washed using 20 mL of DI water and 20 mL ethanol, respectively. Finally, the precipitate was dried in a vacuum oven at 60°C overnight. The sample of $Cu_{0.3\%}/TiO_2$ and $Cu_{0.9\%}/TiO_2$ was prepared by a similar method, except that 100 µL and 200 µL of Cu precursor solution were added, respectively.

Electrocatalyst characterization

Characterization of the electrocatalysts was conducted by a HITACHI SU8020 scanning electron microscope (SEM) and a JEOL JEM-2100F high-resolution transmission electron microscopy (HR-TEM). Inductively coupled plasma atomic emission spectroscopy (ICP-AES) was conducted on a Thermo iCAP RQ. Using Cu-K radiation, an X-ray diffraction (XRD) examination of the electrocatalysts was carried out on a Rigaku D/max-2500 X-ray diffractometer. The samples were subjected to X-ray photoelectron spectroscopy (XPS) utilizing 200 W monochromatic Al K radiation on the Thermo Scientific ESCA Lab 250Xi. The X-ray adsorption spectroscopy (XAS) measurements were performed using a modified flow cell at the 4B9A beamline at Beijing Synchrotron Radiation Facility (BSRF), China. Cu foil, Cu_2O, and CuO were used as references while the data were gathered using a Lytle detector in fluorescent excitation mode. The EXAFS data were processed according to the standard procedures using the Athena and Artemis implemented in the IFEFFIT software packages.

Electrode preparation

The cathode electrode was prepared over a spray-dry method. First, 15 mg of as-prepared electrocatalysts, 1 mL of IPA, and 15 µL of Nafion ionomer solution (5 wt% in IPA) were mixed to prepare the electrocatalyst slurry, followed by ultrasonic treatment for 30 min to form a homogeneous ink. Then, 250 µL of the electrocatalyst slurry was slowly spread onto the carbon paper (TGP-H-60) and dried in the air at room temperature to achieve a uniform electrocatalyst loading of ~1.0 mg cm^{-2}. NF was ultrasonicated with acetone, IPA, and water for 30 min, respectively, and dried in air under room temperature before using as an anode electrode.

Electrochemical study

Electrochemical synthesis of oximes in a flow cell. Electrochemical studies were conducted in an electrochemical flow cell, which was composed of a cathodic chamber and an anodic chamber. An anion exchange member (FumasepFAA-3-PK-130) served to separate the cathodic and anodic chambers. An Ag/AgCl electrode (3.0 mol L^{-1} KCl) and NF were used as the reference electrode and counter electrode, respectively. The electrolysis was conducted through a CHI 660 electrochemical workstation. For performance studies, 5 ml of 1 mol L^{-1} $NaNO_3$ and 50 mmol L^{-1} cyclohexanone aqueous solution was used as catholyte and 1 mol L^{-1} NaOH was used as anolyte. The electrolyte was bubbled with Ar for 30 min under stirring before the electrochemical reaction was started. The surface area of the electrode was 1.4 cm^{-2}. When studying the influence of cyclohexanone to the performance, cyclohexanone of different concentration was added. When studying the concentration of NO_3^-, Na_2SO_4 was used to keep the concentration of all the ions are equal in all studies. The synthesis of cyclohexanone oxime was conducted with 1 mol L^{-1} NO_3^-, 1 mol L^{-1} $NaNO_3$ and 1 mol L^{-1} NaOH.

Product identifications and quantifications

Cyclohexanone oxime. After the electrochemical reaction, 1 mL of catholyte was mixed with 1 mL of saturated NH_4Cl solution to adjust the pH of the solution. Then, the solution was extracted with 10 mL of EA. Reactants and products in EA were identified by gas chromatography-mass spectrometry (GC-MS, Agilent 5977A, HP-5MS capillary column with 0.25 mm of diameter and 30 m of length), and high-resolution GC-MS (Thermo Fisher Scientific, Exactive GC). The analysis of ^{1}H, ^{13}C, and ^{15}N NMR was conducted on Bruker Avance Neo 700 with $CDCl_3$ as the solvent. In the ^{15}N NMR, the reference was CH_3NO_2 + 10% $CDCl_3$, which was calculated as 0 ppm. The quantitative analysis of the products in the extract liquor was conducted using gas chromatography (GC, Agilent 6820) equipped with a flame ionization detector (FID) and HP 5MS/HP-INNOWAX capillary column with 0.25 mm in diameter and 30 m in length. Dodecane was used as the internal standard to quantitative the yields of electrochemical reactions. The calibration curve was established by preparing and measuring the peak area ratio between cyclohexanone oxime and dodecane. Several standard EA solution samples with the known concentration of cyclohexanone oxime were used. The concentration of cyclohexanone oxime of unknown extract liquor samples was then calculated through the calibration curve.

The Faradaic efficiency (FE) and yield rate of cyclohexanone oxime were calculated as follows:

$$FE_{cyclohexanone\ oxime} = \frac{nFcV}{Q}$$

$$Formation\ rate_{cyclohexanone\ oxime} = \frac{McV}{St}$$

Where n is the electrons transferred in per mole reaction of electrosynthesis of cyclohexanone oxime, F is the Faraday constant, c is the concentration of the products, V is the volume of the

electrolyte used in the reaction, Q is the total number of charges transferred in the reaction, M is the molecular mass of the product, S is the area of the electrode in the flow cell, and t is the reaction time.

The gaseous products were collected in gas bags and analyzed by gas chromatography (GC, HP 4890D) with Ar as the load gas.

Long-established colorimetric methods were utilized to quantify the concentration of NH_3 and NO_2^- in the current study with some modifications[1]. All the samples were taken for UV-vis absorption measurement on Perkin Elmer, Lambda 1050+ within 2 h after preparation. The salicylate method was used to quantify NH_3 in the catholyte, enabled by the transformation of NH_3 to indophenol blue through a chemical reaction among NH_3, salicylate, and hypochlorite. Solutions A, B, and C were prepared as follows. Solution A: 0.32 mol L^{-1} sodium hydroxide + 0.4 mol L^{-1} sodium salicylate aqueous solution. Solution B: 0.75 mol L^{-1} NaOH + NaClO (active chlorine: ~4.5%) aqueous solution. Solution C: 10 mg ml^{-1} $C_5H_4FeN_6Na_2O_3$ aqueous solutions. To quantify the concentration of NH_3 in the catholyte, 3 mL of the aqueous sample solution, 500 µL of Solution A, 50 µL of Solution B, and 50 µL of Solution C were sequentially added to a sample tube. The fresh electrolyte was used to prepare the blank sample for UV-vis measurement as the baseline. The calibration curve was established by preparing and measuring the absorbance at 675 nm of several standard solution samples with the known concentration of NH_4^+ in the typical electrolyte of electrosynthesis of oximes. The concentration of NH_3 in unknown samples was calculated through the absorbance at 675 nm and the calibration curve.

The FE of NH_3 was calculated as follows:

$$FE_{NH_3} = \frac{nFcV}{Q}$$

where n is the electrons transferred in pre mole reaction of generation of NH_3, F is the Faraday constant, c is the concentration of the NH_3, V is the volume of the electrolyte, and Q is the total number of charges transferred in the reaction.

Griess test was used to quantify NO_2^- in the catholyte, in which azo compound with pink color could form through the reaction among NO_2^-, 4-aminobenzenesulfonamide, and N-(1-naphthyl) ethylenediamine. Solution α and β were prepared for further use. Solution α: 10 g L^{-1} of 4-aminobenzenesulfonamide aqueous solution. Solution β: 1 g L^{-1} N-(1-naphthyl)ethylenediamine dihydrochloride aqueous solution. When qualifying the concentration of NO_2^- in an aqueous sample, 200 µL of solution α and 200 µL of solution β were added to the sample at a 10-minute interval. The fresh electrolyte was also used to prepare the blank sample for UV-vis measurement as the baseline. The calibration curve was established by preparing and measuring the absorbance at 540 nm of several standard solution samples with the known concentration of NO_2^- in the typical electrolyte of electrosynthesis of oximes. The concentration of NO_2^- of unknown samples was calculated through the absorbance at 540 nm and the calibration curve.

The Faradaic efficiency (FE) of NO_2^- was calculated as follows:

$$FE_{NO_2^-} = \frac{nFcV}{Q}$$

where n is the electrons transferred in per mole reaction of generation of NO_2^-, F is the Faraday constant, c is the concentration of the NO_2^-, V is the volume of the electrolyte, and Q is the total number of charges transferred in the reaction.

Plasma air oxidation

The air oxidation plasma reaction system was composed of a stainless-steel rotating gliding plasma jet reactor, a gas feeder, mass flow controllers, a DC power supply, and a current-limiting resistor. The reactor had a cylindrical anode inserted into a cylindrical cathode, with a non-thermal plasma ignited in the cavity and expelled through a nozzle-designed outlet via the gas flow. The reactants used were a mixture of N_2 and O_2 (model air) to produce NO_x products. 3.0 mL of sample solution was added to the test tube, and then 100 µL of 5.0 M HCl solution was added. After shaking well and standing for 10 min, the concentration of NO_3^- was measured by UV-Vis in the wavelength range of 200 ~ 300 nm. The standard curve for NO_3^- determination was plotted with the difference of absorbance values at 220 nm and 275 nm as vertical coordinates and NO_3^- concentration as horizontal coordinates. The concentration of NO_2^- was as above-mentioned.

Isotope-labeled experiments

The isotope-labeled electrochemical synthesis of oximes was performed on the 0.6% Cu/TiO_2 electrode with 1 mol L^{-1} $Na^{15}NO_3$ and 50 mmol L^{-1} cyclohexanone aqueous solution at -1.6 V versus Ag/AgCl for 1 h. The ^{15}N-cyclohexanone oxime was not commercially available. The home-made standard sample of ^{15}N-cyclohexanone oxime was synthesized by mixing 0.05 mol L^{-1} ^{15}N-$(NH_2OH)_2H_2SO_4$ with 0.05 mol L^{-1} cyclohexanone aqueous solution and stirring for 30 min. The $CDCl_3$ extract liquor of the solution was used as the standard sample after being detected by GC-MS.

DEMS experiments

Differential electrochemical mass spectrometry (DEMS) experiments were conducted on Shanghai Linglu QAS 100. A typically optimized electrolyte and 0.6% Cu/TiO_2 catalyst were used in the test. Ar was used as the loaded gas in the whole experiment to avoid the influence of other reactions.

In situ FTIR experiments

In situ FTIR experiments were conducted on Thermo Scientific Thermo 8700. A typically optimized electrolyte and 0.6% Cu/TiO_2 catalyst were used in the experiment with Ar atmosphere. In the experiments in the D_2O solvent, the gas path, electrochemical cell, and reference electrode were washed with D_2O several times to remove H_2O. The solution in the reference electrode was also changed to 3M KCl in D_2O.

Density functional theory (DFT) calculations

Vienna ab initio simulation package (VASP) was used to Spin-polarized density functional theory (DFT) calculations.[2] The projector augmented wave (PAW) pseudopotential for core electrons and the generalized gradient approximation (GGA) in the form of Perdew-Burke-Ernzerhof (PBE) for the exchange correlation potentials were adopted.[3, 4] The c axis of 25 Å was stetted to avoid the interactions of catalysts between adjacent images. In addition, a cut-off energy of 450 eV was used. The atoms were fully relaxed until the energy convergence reached 0.0001 eV. Van der Waals (vdW) interaction was considered at the DFT-D3 level as proposed by Grimme.

Table S1. Control experiments to reveal the electrosynthesis pathway of cyclohexanone oxime.

Entry	C-source	N-source	Electrolysis	Cyclohexanone oxime	FE (%)	Formation rate (mg h^{-1} cm^{-2})
1	N/A	NO_3^-	Yes	No	N/A	N/A
2	Cyclohexanone	N/A	Yes	No	N/A	N/A
3	Cyclohexanone	NO_3^-	Yes	Yes	50.0	20.1
4	Cyclohexanone	NO_2^-	Yes	Yes	47.8	20.0
5	Cyclohexanone	NO	Yes	Yes	7.6	19.8
6	Cyclohexanone	NH_2OH	Yes	Yes	N/A	20.1
7	Cyclohexanone	NH_2OH	No	Yes	N/A	20.1

Table S2. FE and formation rate of different oxime molecules.

Substrate	Product	FE (%)	Selectivity (%)	Found molecular mass
Cyclohexanone	Cyclohexanone oxime	50.0	>99.9	113.1
Cyclopentanone	Cyclopentanone oxime	47.8	>99.9	99.1
Diethyl ketone	Diethyl ketone oxime	38.2	>99.9	101.1
Benzaldehyde	Benzaldehyde oxime	17.2	78.3	121.0
Acetone	Acetone oxime	21.7	65.7	87.1

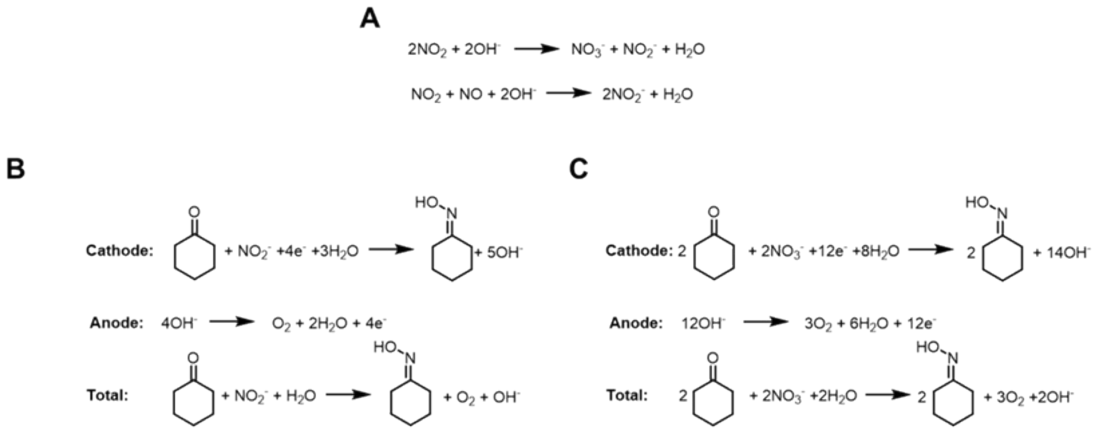

Figure S1. Chemical reactions involved in the synthesis of cyclohexanone oxime.

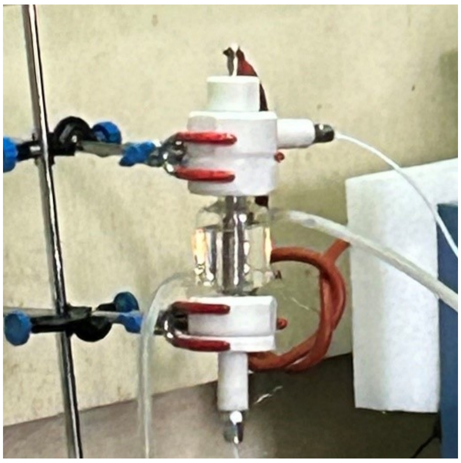

Figure S2. Optical graph of home-made plasma air oxidation reaction unit.

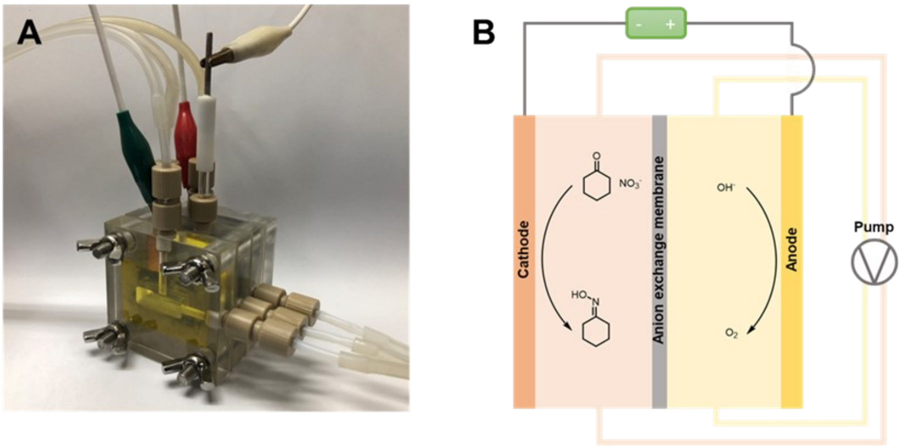

Figure S3. (A) Optical graph and (B) illustration of applied flow cell.

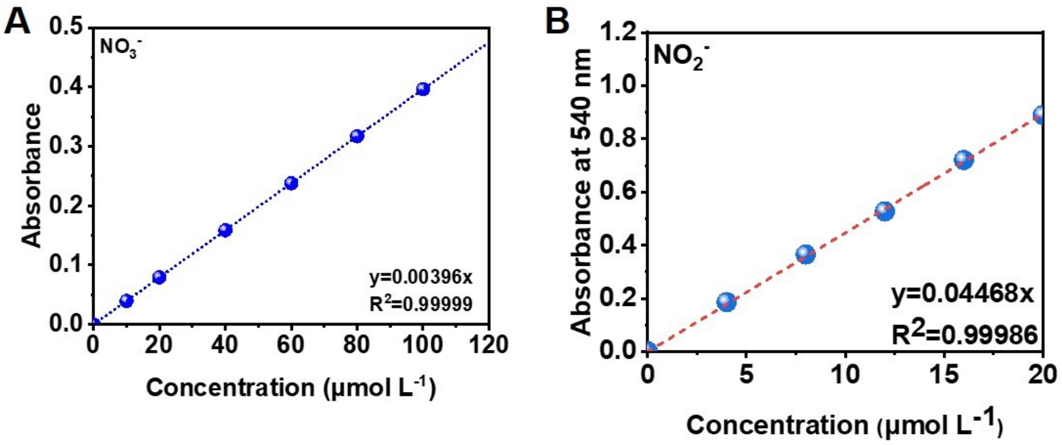

Figure S4. The standard curve to quantitative (A) NO_3^- and (B) NO_2^-.

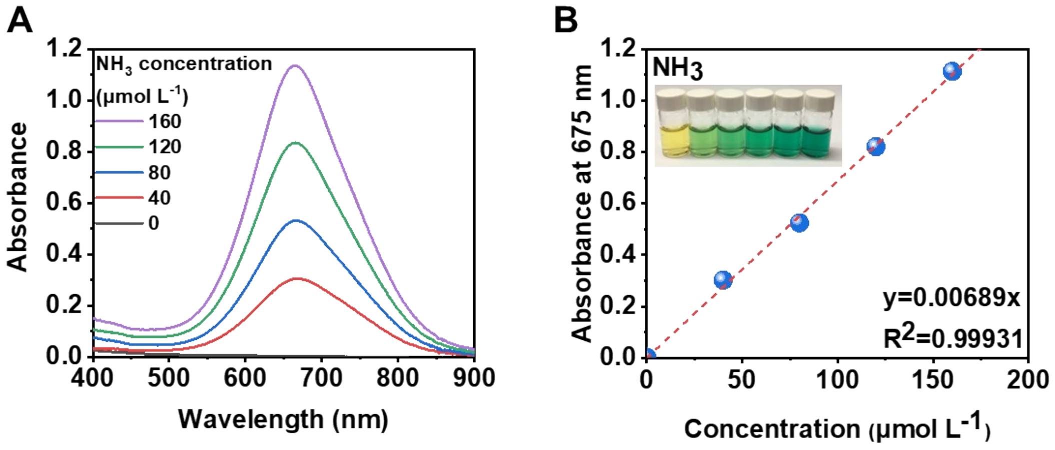

Figure S5. The standard curve to quantitative NH_3.

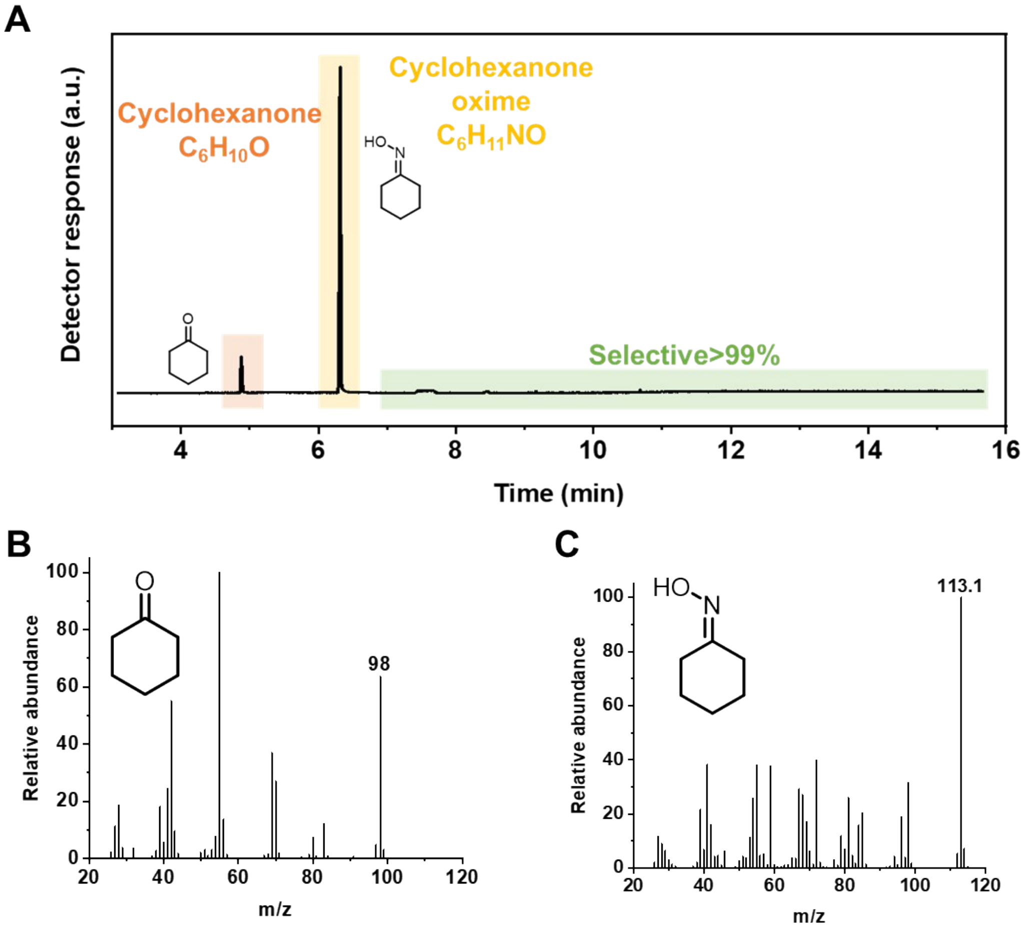

Figure S6. (A) GC-MS detection of the products. MS graph of detected (B) cyclohexanone and (C) cyclohexanone.

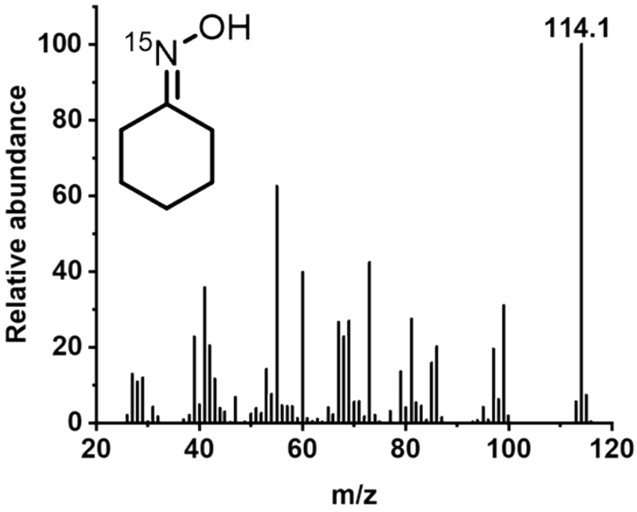

Figure S7. MS graph of detected ^{15}N-cyclohexanone oxime.

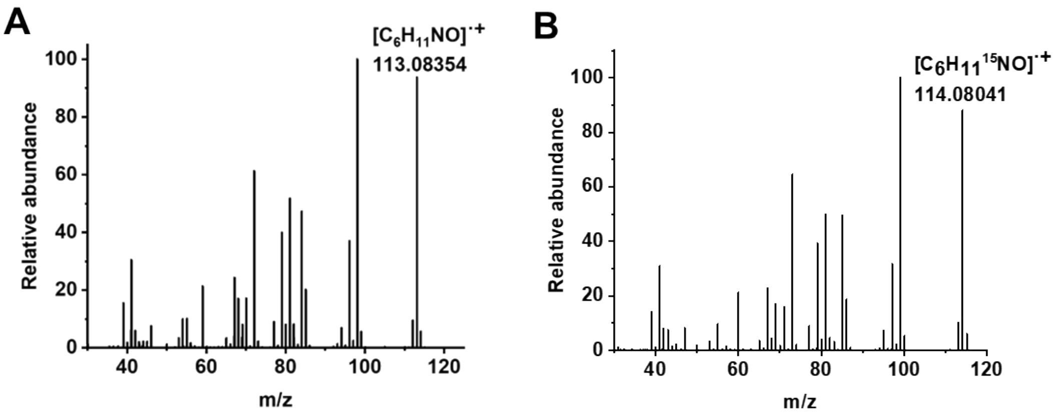

Figure S8. Full MS graph of (A) ^{14}N and (B) ^{15}N cyclohexanone oxime from HR-GC-MS.

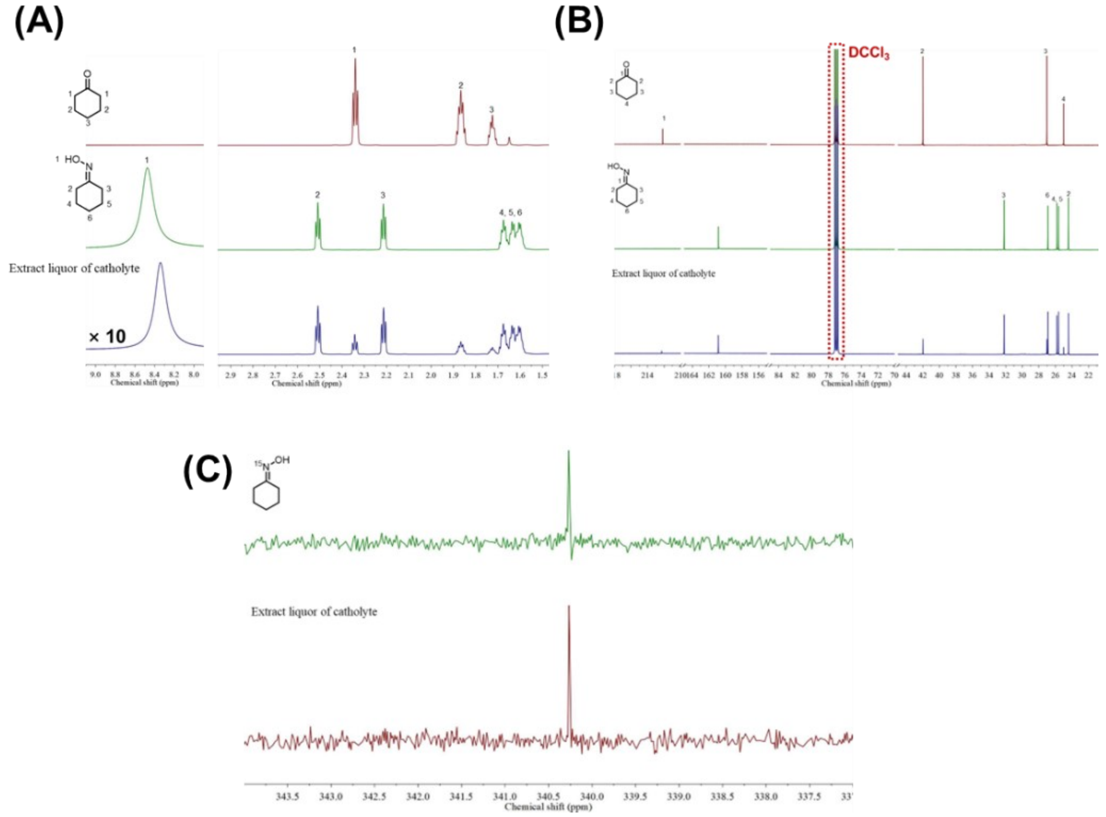

Figure S9. Full graph of (A) ^{1}H NMR, (B) ^{13}C NMR, and (C) ^{15}N NMR of standard samples and extract liquor of catholyte.

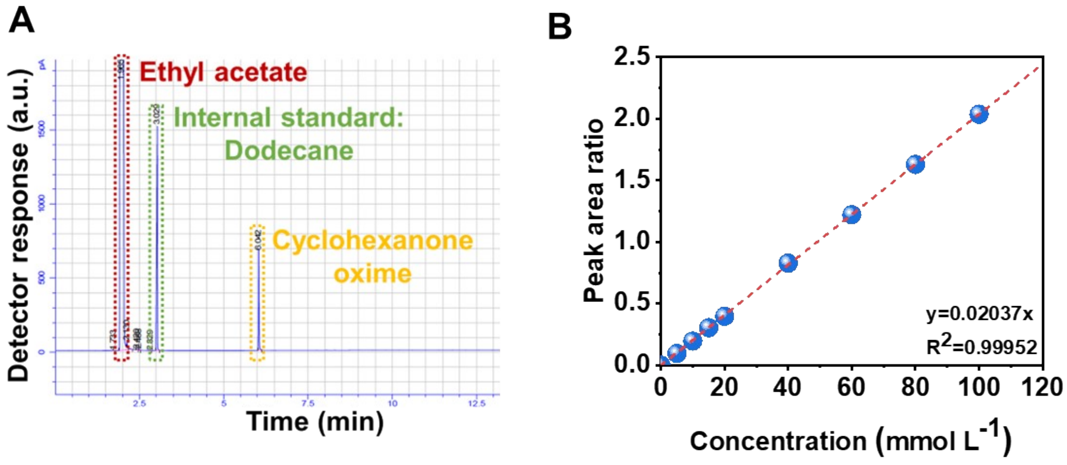

Figure S10. The standard curve to quantitative cyclohexanone oxime.

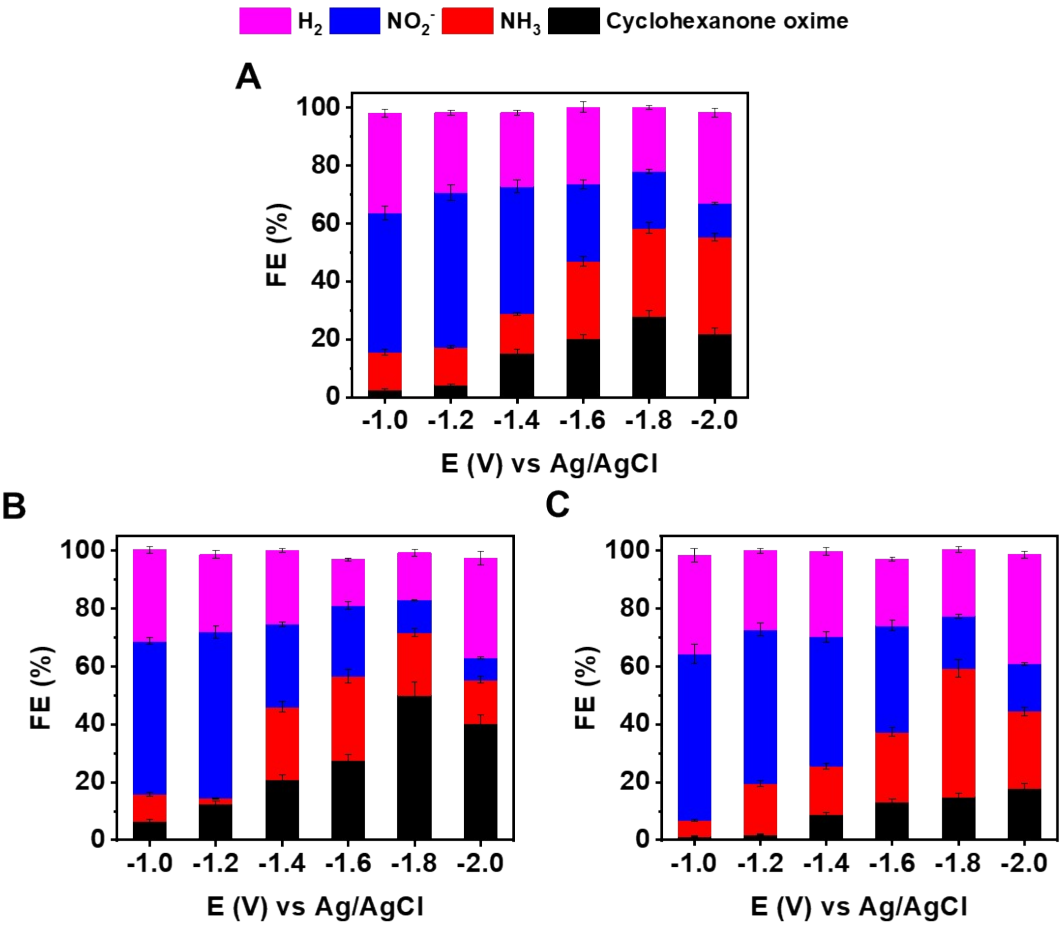

Figure S11. Electrochemical performance of (A) 0.3% Cu/TiO_2, (B) 0.6% Cu/TiO_2, and (C) 0.9% Cu/TiO_2.

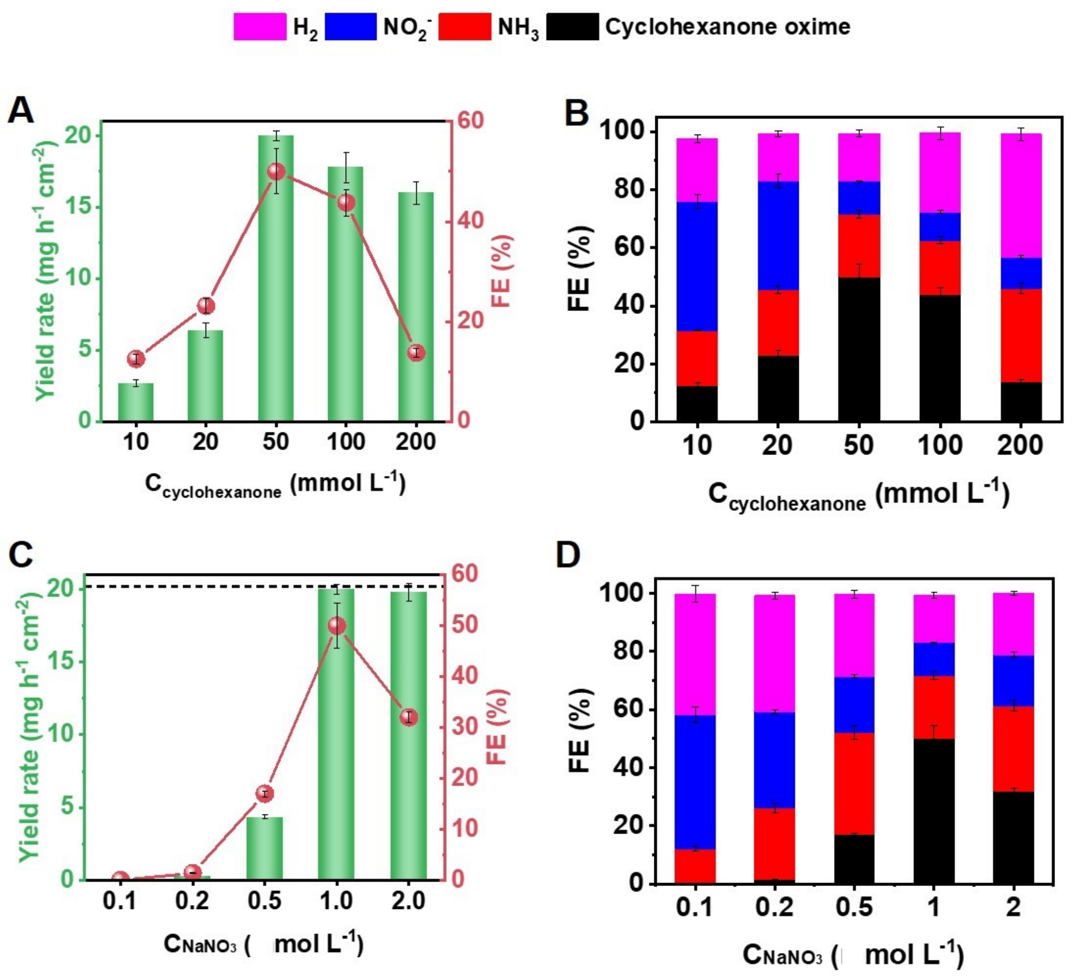

Figure S12. Electrochemical performance with electrolyte with different (A)-(B) cyclohexanone and (C)-(D) $NaNO_3$ concentration. The dotted line shows the yield rate of cyclohexanone at 100% conversion.

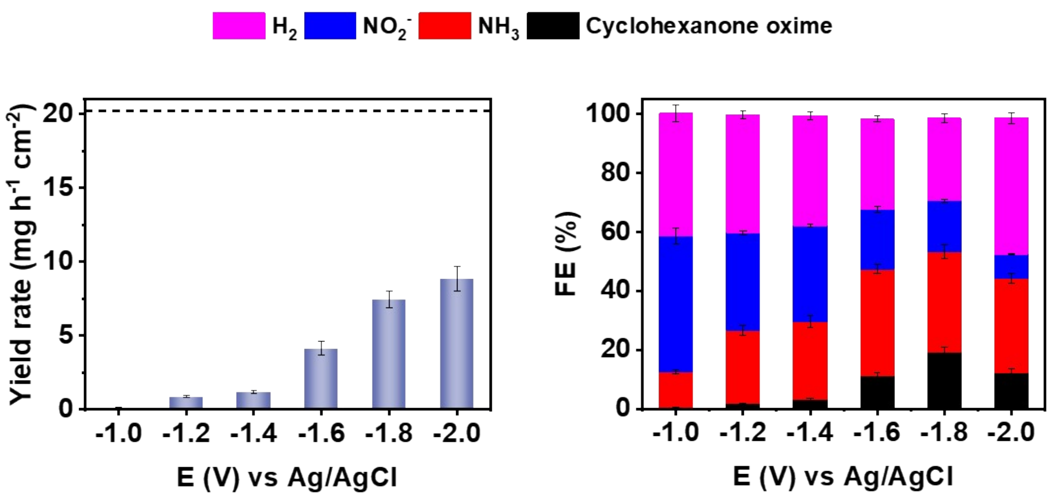

Figure S13. Electrochemical performance of bulk TiO_2 catalyst.

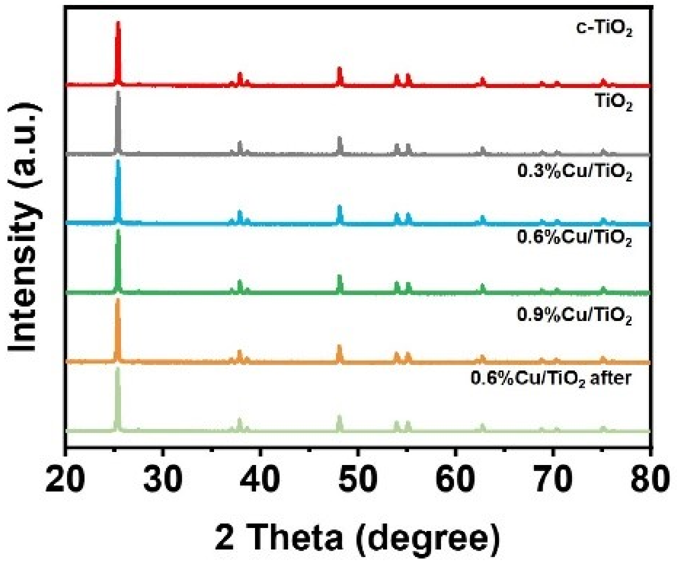

Figure S14. PXRD of different catalysts.

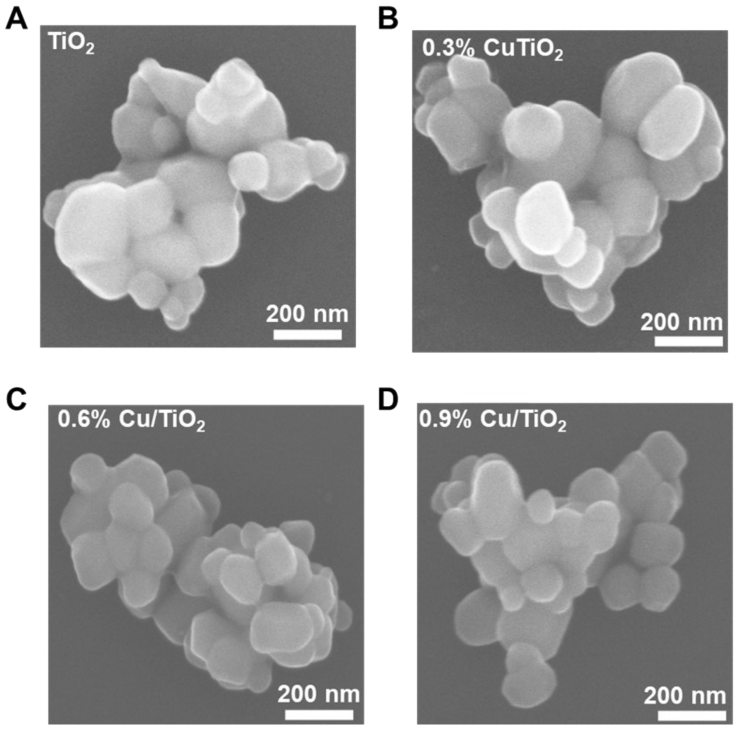

Figure S15. SEM image of different catalysts. (A) TiO_2, (B) 0.3% Cu/TiO_2, (C) 0.6% Cu/TiO_2, and (D) 0.9% Cu/TiO_2.

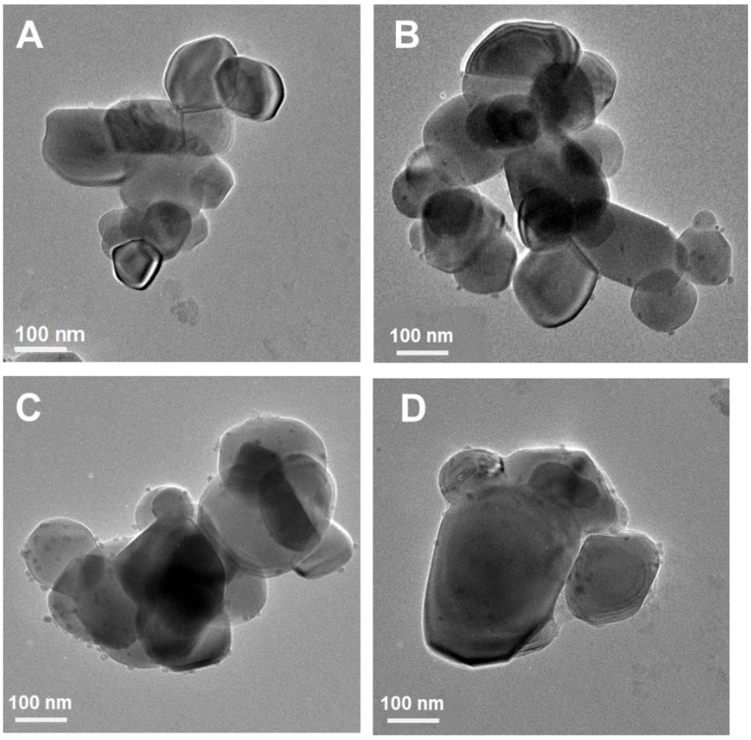

Figure S16. HRTEM image of different catalysts. (A) TiO_2, (B) 0.3% Cu/TiO_2, (C) 0.6% Cu/TiO_2, and (D) 0.9% Cu/TiO_2.

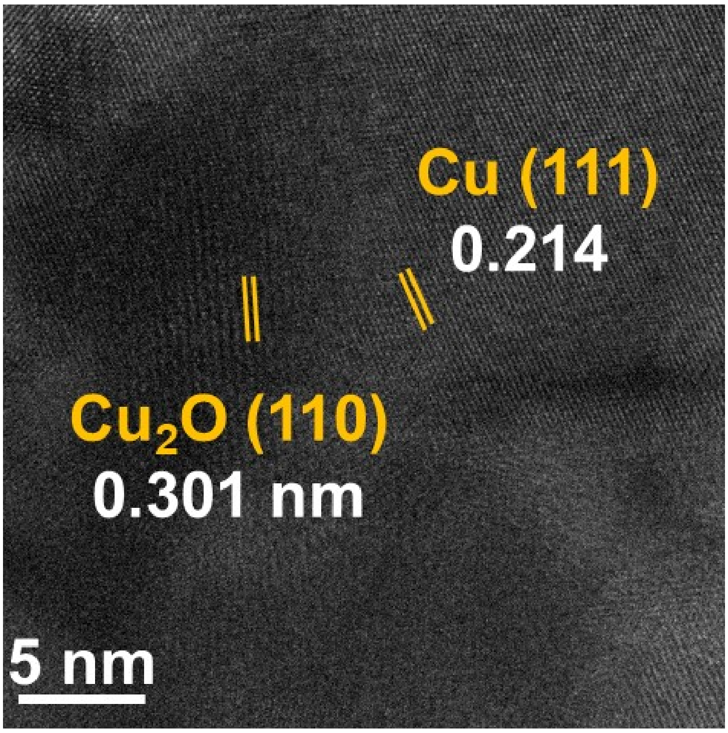

Figure S17. HRTEM image of 0.6% Cu/TiO_2.

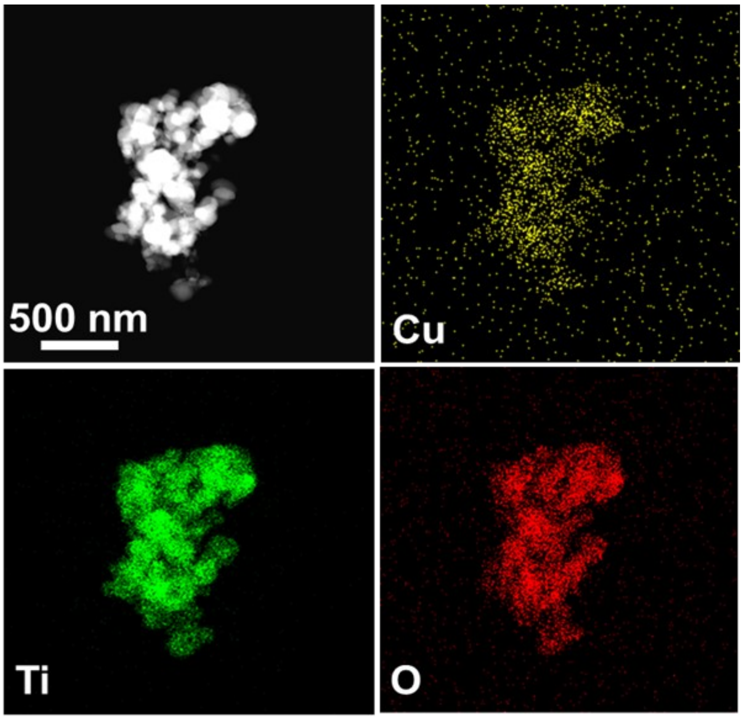

Figure S18. Energy dispersive spectroscopy (EDS) mapping of 0.6% Cu/TiO_2. Yello, green, and red present Cu, Ti, and O, respectively.

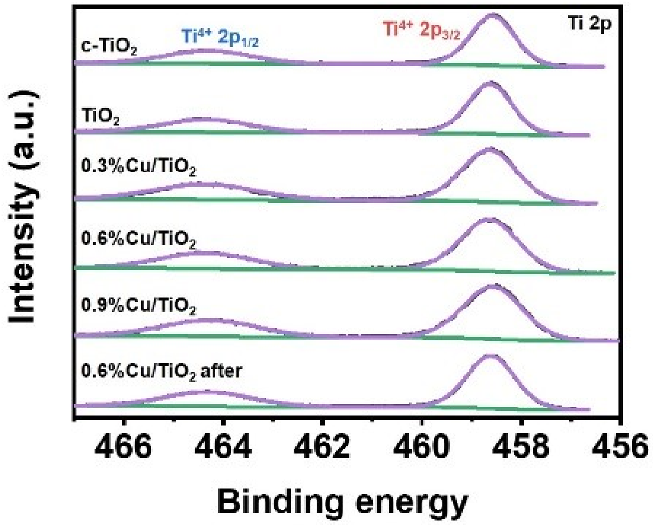

Figure S19. Ti 2p XPS image of different catalysts.

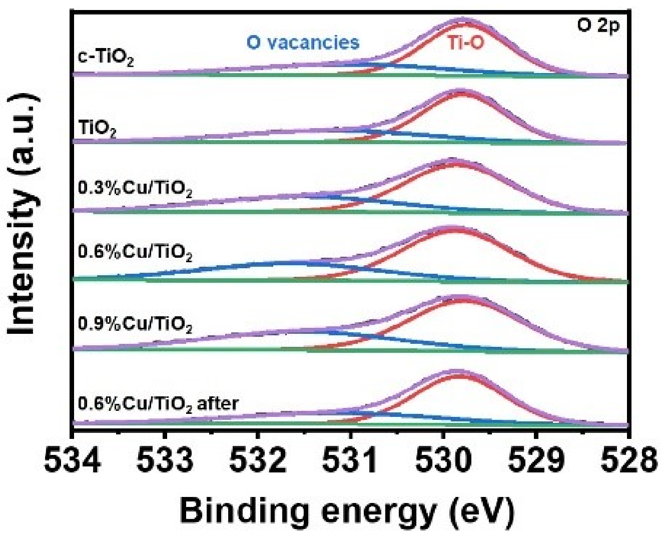

Figure S20. O 2p XPS image of different catalysts.

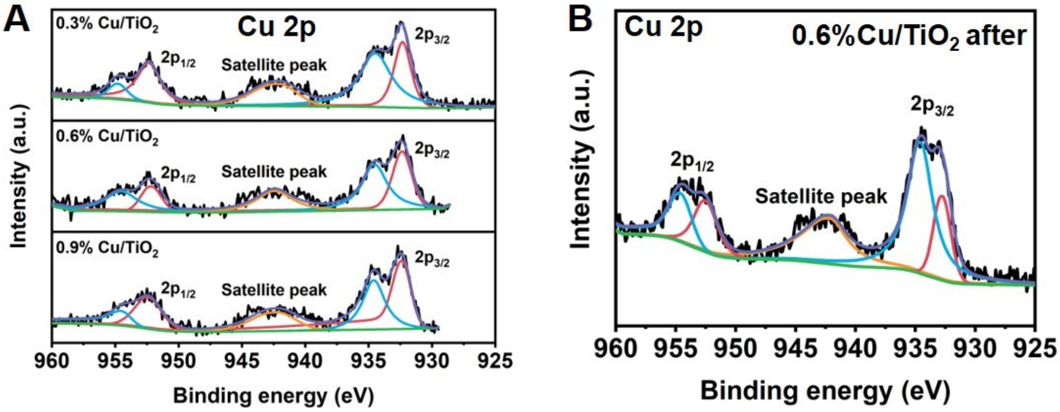

Figure S21. Cu 2p XPS image of (A) different catalysts before electrolysis and (B) 0.6% Cu/TiO_2 catalysts after electrochemical reaction.

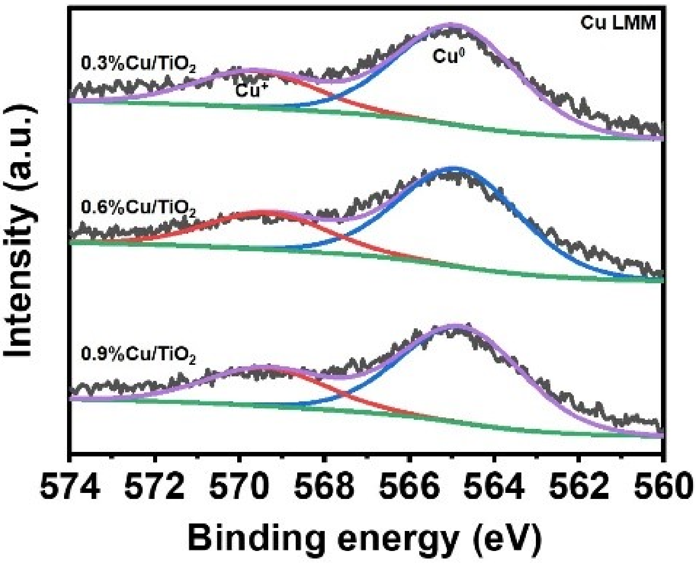

Figure S22. Cu LMM XPS image of different catalysts.

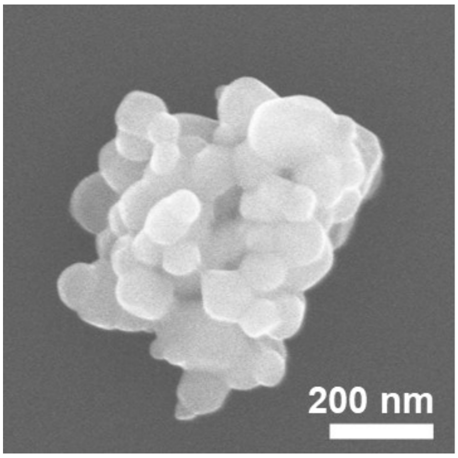

Figure S23. SEM image of 0.6% Cu/TiO_2 after electrolysis.

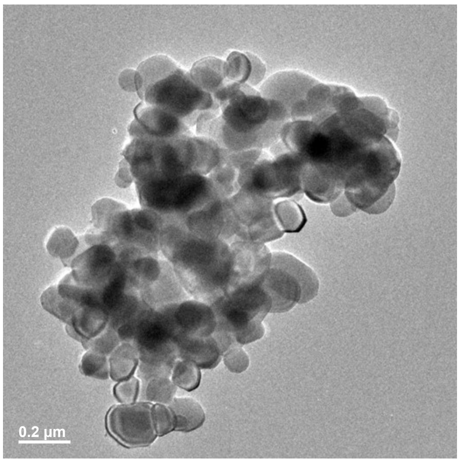

Figure S24. TEM image of 0.6% Cu/TiO_2 after electrolysis.

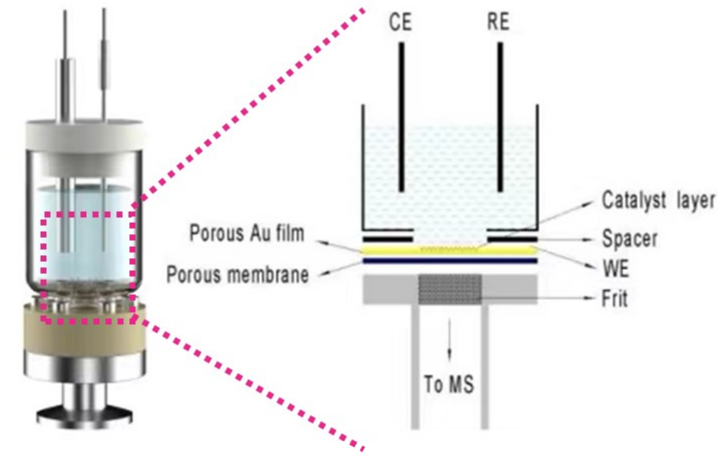

Figure S25. Electrochemical cells using in online DEMS experiments.

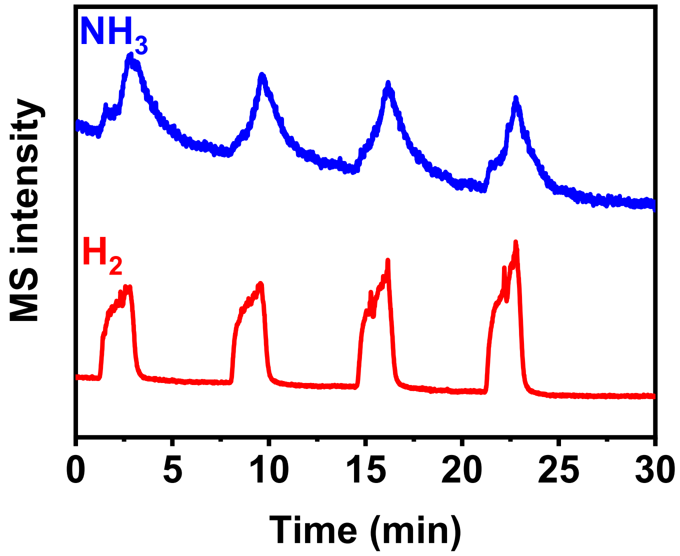

Figure S26. Signals of byproducts in online DEMS.

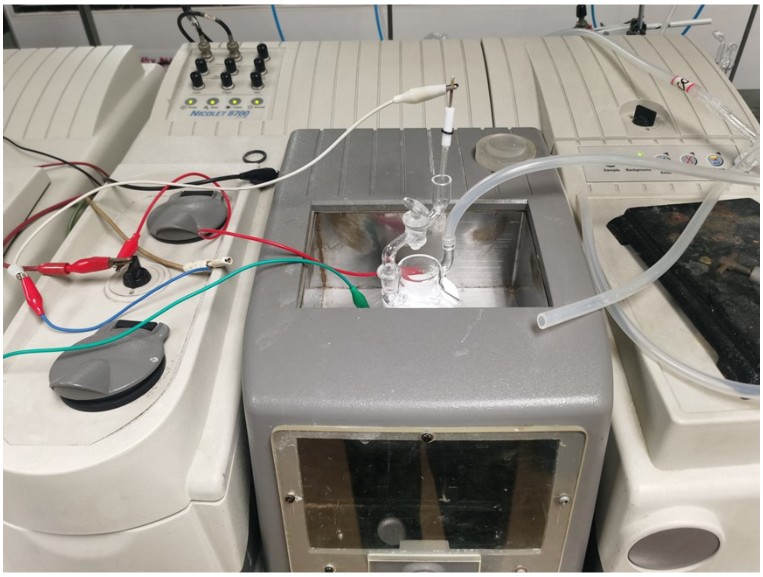

Figure S27. Electrochemical cells using in *in situ* FTIR experiments.

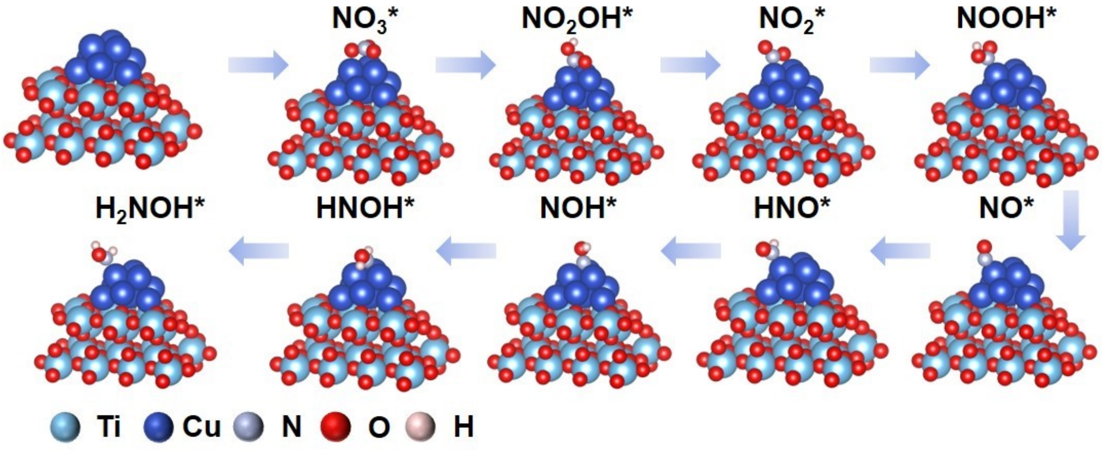

Figure S28. Reaction pathway on Cu(111)/TiO_2(101) model according to the optimized configurations with adsorbed in-termediates. Bule, purple, red, and pink balls stand for Ti, C, H, and H, respectively.

References

1. Y. Wu, Z. Jiang, Z. Lin, Y. Liang and H. Wang, Direct electrosynthesis of methylamine from carbon dioxide and nitrate, *Nat. Sustain.*, 2021, **4**, 725-730.
2. G. Kresse and J. Furthmüller, Efficiency of ab-initio total energy calculations for metals and semiconductors using a plane-wave basis set, *Comp. Mater. Sci.*, 1996, **6**, 15-50.
3. P. E. Blöchl, O. Jepsen and O. K. Andersen, Improved tetrahedron method for Brillouin-zone integrations, *Physical Review B*, 1994, **49**, 16223-16233.
4. J. P. Perdew, K. Burke and M. Ernzerhof, Generalized Gradient Approximation Made Simple, *Phys. Rev. Lett.*, 1996, **77**, 3865-3868.
